# Fertilizers and Human Health—A Systematic Review of the Epidemiological Evidence

**DOI:** 10.3390/toxics12100694

**Published:** 2024-09-26

**Authors:** Christos F. Tagkas, Evangelos C. Rizos, Georgios Markozannes, Maria A. Karalexi, Lydia Wairegi, Evangelia E. Ntzani

**Affiliations:** 1 Evidence-Based Medicine Unit, Department of Hygiene and Epidemiology, School of Medicine, University of Ioannina, 45110 Ioannina, Greece; c.tagkas@uoi.gr (C.F.T.); g.markozannes@uoi.gr (G.M.); marykaralexi@windowslive.com (M.A.K.); 2 School of Health Sciences, University of Ioannina, 45110 Ioannina, Greece; vagrizos@uoi.gr; 3 Independent Researcher, Nairobi P.O. Box 16768, Kenya; wanjawairegi@gmail.com; 4 Biomedical Research Institute, Foundation for Research and Technology-Hellas (FORTH), 45110 Ioannina, Greece; 5 Department of Health Services, Policy and Practice, School of Public Health, Brown University, Providence, RI 02912, USA

**Keywords:** fertilizers, human, health, systematic review, epidemiology

## Abstract

Background: Fertilizers are widely used to supply nutrients to crops, thereby increasing yields and soil fertility. However, the effects of their production and application on human health through occupational, residential, and environmental exposure remain unclear. Objective: To conduct a systematic review of epidemiological studies on the association between exposure to fertilizers and health-related outcomes. Methods: We searched in PubMed, Scopus, and Web of Science for cohort, case-control, cross-sectional, and ecological studies (up to May 2024) related to exposure to fertilizers and any reported human health endpoints across all age groups, without language or geographical limitations. Data were extracted for population and study characteristics, type of fertilizer used, exposure assessment, sample size, outcome and its definition, effect estimate, and quality characteristics from the eligible studies, and they were descriptively synthesized. Results: We found 65 eligible publications, with 407 postulated associations. Forty-six publications (321 associations) assessed exposure to inorganic fertilizers, and nineteen studies (93 associations) assessed organic fertilizers. Exposure assessed was related to occupation, residence, and/or proximity. The assessed outcomes were diverse, with considerable harmonization challenges. Inorganic fertilizers have been associated with an increased risk of cancerous outcomes in a small number of studies with methodological limitations and low replication validity, while organic fertilizers have been associated with infections and diarrhea. Conclusions: The epidemiological evidence suggests possible associations between inorganic fertilizers with solid organ tumors and hematological malignancies and organic fertilizers with infections and diarrhea. However, the available evidence is limited, and heterogeneity prevails. Further research is needed to enlarge the evidence base and increase the replication validity and robustness of the results.

## 1. Introduction

As the world’s population steadily expands and available arable land diminishes, the issue of global food production becomes increasingly important [1]. Fertilizers, derived from mineral, synthetic, and organic sources, have played a crucial role in modern agriculture by significantly increasing crop yields, thus ensuring essential human nutrition, global food security, crop quantity and quality, and sustainable soil management. According to the Food and Agriculture Organization, fertilizer can be a chemical or natural substance or material that is used to provide nutrients to plants, usually via application to the soil, but also to foliage or through water in rice systems, fertigation or hydroponics or aquaculture operations [2]. Their aim is to supply nutrients to plants, thereby increasing soil fertility and crop yields, and to supplement and restore soil nutrients to maintain good soil condition.

Although the use of fertilizers in agriculture has increased over the years, information on their impact on public health and the environment is limited. Fertilizers are an essential tool for soil management, but their use may result in an increase in contamination of soils, air, and water either from the elements of the fertilizers or their contaminants, such as toxic trace elements for inorganic and pathogens for organic fertilizers. Along with the application of inorganic fertilizers, levels of nitrogen compounds in water, air, and soil have doubled over the past 100 years, affecting plants, animals, and humans [3]. Recent projections for fertilizer use indicate an evolving landscape influenced by various market and environmental factors. According to the International Fertilizer Association (IFA), global primary nutrient sales, including nitrogen, phosphorus, and potassium, are expected to continue growing, albeit at a slower rate. By 2024, the global demand for fertilizers is projected to reach approximately 263 million tonnes, reflecting an average annual growth rate of about 0.9% from 2020 [4].

The report of the third session of the United Nations Environmental Assembly highlighted the lack of available data on the impact of fertilizers on both human health and the environment [5]. Conducting epidemiological research on fertilizers presents unique challenges because of their frequent concurrent use with insecticides, herbicides, and fungicides, which complicates the ability to isolate and attribute specific health effects to individual substances. This overlapping usage creates confounding variables that make it difficult to disentangle the distinct impacts of fertilizers from those of other agrochemicals. In this review, we sought to systematically assess the state of the evidence regarding the available epidemiological data on the association between fertilizer use and human health.

## 2. Materials and Methods

### 2.1. Search Strategy

We searched PubMed, Scopus, and Web of Science up to May 2024 using two distinct and complementary search algorithms: (“fertile*” OR “chemical fertilizer” OR “synthetic fertilizer” OR “organic fertilizer” OR “organic inputs” OR “mineral fertilizer” OR “organic manure” OR “manure”) (limited to “human”), complemented by an algorithm capturing the biomedical literature related to the use of sewage sludge as fertilizer, using the terms: ((“waste” OR “sewage sludge” OR “biosolids” OR “amendment”) AND (“farming” OR “crop*” OR “soil”)) across all age groups (from newborns to adults). Our search was not restricted by outcome-related terms so as to map the outcomes studied related to fertilizer exposure and identify emerging outcomes. Title and abstract screening were performed by two independent researchers (C.F.T. and E.C.R), and discrepancies were resolved by a third arbitrator (E.E.N.). The researchers used the online machine-learning tool *Abstrackr* (It is a free, open-source, web-based application created by the Center for Evidence-Based Medicine (CEBM) at Brown University, located in Providence, Rhode Island, USA [6] for this purpose.

### 2.2. Study Eligibility and Selection

**Figure 1:**
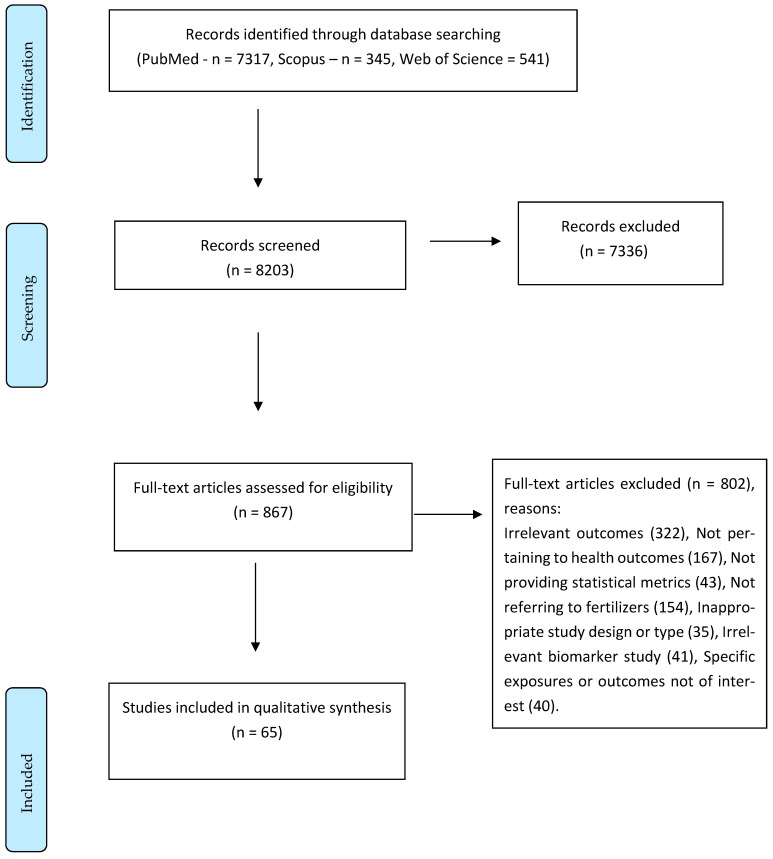
Systematic review PRISMA flow chart.

We included observational studies (cohort, cross-sectional, case-control, and ecological studies) that evaluated the association between exposure to any fertilizer and any health-related outcome in humans. No restrictions to language or geographical region were applied. Case reports, case series, narrative reviews, modeling studies, and editorials, as well as animal or in vitro studies, were excluded. We excluded studies that assessed acute or accidental exposure to fertilizers, studies that did not examine outcomes related to human health, and studies that linked contact to fertilizers with a specific biological pathway via assays or biomarkers that were not clinically validated. We further excluded studies with no quantitative information on the reported outcomes, such as effect estimates and 95% confidence interval (CI), counts and sample sizes, crude or adjusted means, or standard deviation (SD). Figure 1 shows the PRISMA flow chart of our study process, and Table 1 shows the PECO statement [7] of our research endeavor.

### 2.3. Exposure and Outcome Definitions

Exposure to fertilizers was considered mainly occupational or residential. Exposure assessment was defined through information coming from study participants (using self-administrated questionnaires, interviewer-administrated questionnaires, job-exposure matrixes, or occupational history), residential status data (regarded as proximity to fertilizers’ exposure), governmental registries of fertilizers’ application, or specific biomarkers. As for the health-related outcomes, we assessed all types of outcomes related to human health, including clinical outcomes (e.g., disease incidence), clinically applied tools (such as neurocognitive scales), or established biomarkers (such as liver enzymes).

### 2.4. Data Extraction

We created a simple and user-friendly tool in excel for recording all the available evidence of eligible studies. We recorded the name of the first author, the journal, and the year of publication from each publication. In addition, we recorded the type of fertilizer exposure and the number of outcomes under investigation. Therefore, one study may provide evidence for more than one assessment/association each time.

The list of extracted outcomes was finalized following pilot data extraction in a random sample of ten eligible studies in order to identify areas that needed refinement or additional data that needed to be extracted. We gathered extensive details, including PubMed ID, first author, publication journal and year of publication, study location, sample size for cases and controls, recruitment and exposure periods, and follow-up duration. Information was also extracted on the type of epidemiological study, cohort details, and population demographics, such as age and gender, exposure types, and timing of these exposures. For exposure assessment, we collected data on fertilizer types, application methods, exposure modes, co-exposures with other chemicals, the methods used for assessing exposure, definitions, frequency, magnitude of exposure, and access to storage sites of fertilizers.

Data extraction related to health outcomes included their definition as described in the text and the relevant disease category. We also extracted the association measure used, the relevant point estimate and uncertainty thereof, and any confounding factors that were adjusted for. Finally, we obtained information pertaining to the methodological and quality characteristics of the eligible studies. Data extraction was performed by a single investigator (C.F.T.), cross-checked by another (E.C.R.), and any discrepancies were resolved by a third researcher (E.E.N.).

### 2.5. Quality of the Assessed Evidence

We appraised the methodological aspects of the included studies and the risk of bias conferred thereof by using elements of the RTI item bank [8], which is a practical and validated tool for evaluating the risk of bias and precision of observational studies, interventions, or exposures included in systematic reviews. The quality assessment of these studies is available in Table 2 and Table 3 and in Supplementary Table S1 This systematic review is reported following the PRISMA guidelines, and the PRISMA-P checklist is available in Supplementary Table S4.

### 2.6. Evidence Synthesis

Considering the heterogeneity of the assessed exposures and outcomes, we opted for a descriptive synthesis of the results. We organized the evidence synthesis by adopting two approaches: grouping studies per disease entity and the general category of the fertilizer under study (inorganic versus organic). Following this categorization, diseases with a shared pathophysiological background were further combined as appropriate. Additionally, we considered together the different exposure windows (preconception, conception, childhood, and adulthood) and types of exposure (during production, which is expected to be high and long term; during application, which is expected to be lower to moderate and intermittent; and exposure due to residential vicinity where fertilizers are applied). In the main text of the manuscript, a narrative summary of the disease entities assessed in more than three studies is provided. The comprehensive study information across the entire evidence base is presented in Table 2 and Table 3, which include data exclusively based on specific clinical entities rather than symptoms. Additionally, the Supplementary materials contains all extracted data from the original studies, providing a complete overview of the analyzed evidence. 

## 3. Results

### 3.1. Evidence Base Overview-Study Characteristics-Methodological Assessment

Among the 867 full-text publications eligible for further scrutiny out of 8203 citations initially retrieved, we finally included 65 publications [9,10,11,12,13,14,15,16,17,18,19,20,21,22,23,24,25,26,27,28,29,30,31,32,33,34,35,36,37,38,39,40,41,42,43,44,45,46,47,48,49,50,51,52,53,54,55,56,57,58,59,60,61,62,63,64,65,66,67,68,69,70,71,72,73]. Studies were published from 1981 to 2020 and included various populations across the globe, mostly from the USA, China, Italy, and Norway. Continent-wise, America and Europe represented the vast majority of studies, followed by Asia and Africa. One-fifth of participants were recruited before the 1980s, whereas one-fourth of participants were recruited during the 2000s. Regarding study design, case-control studies were the majority (51%), followed by cross-sectional and cohort studies (23% and 19%, respectively), ecological studies (6%), and two (3%) were follow-up studies of cancer mortality among fertilizer plant workers, lasting 17–61 years. Regarding gender, 18% of the studies examined only male and 5% only female participants, while the rest of them either did not provide this information or, when provided, the population of interest was gender balanced.

In 39 studies (60%), participants were occupationally exposed to fertilizers; in 15% of these studies, people resided near regions where fertilizers were used, and in 12%, both occupational and residential exposure was mentioned. In forty-six studies (71%), participants were exposed to inorganic fertilizers; in sixteen studies, they were exposed to organic fertilizers; in the three remaining studies, they were exposed to both types of fertilizers and biosolids. Eight studies examined exposure to fertilizers during their production. Four studies referred to exposure to fertilizers due to residential proximity to fertilizer factories. The rest of the studies investigated either direct exposure to fertilizers from application or environmental exposure to fertilizers. Adulthood was the most frequent (56%) exposure window, followed by childhood (22%) and preconception or pregnancy (12%). Investigators documented exposure by using questionnaires in approximately 60% of the studies, by specifically asking participants for occupational or residential history (23% and 9%, respectively), by using job matrices (2%), or by air markers to quantify the worker’s exposure in fertilizer plants (5%). We found no study referring to biomarkers.

Among the 407 associations found, solid organ tumors and hematological malignancies were the most commonly studied outcomes (23% and 16%, respectively), followed by neurological (12%), infectious (9%), and respiratory diseases (9%).

The methodological assessment showed that 25% of the associations derived from prospective studies, and 85% of the associations provided details about inclusion and exclusion criteria. A total of 81% of the associations used a validated method to appraise the outcome, and effect estimates were adjusted for multiple risk factors in 67% of the proposed associations. In half of the pertinent studies, there was a balance between cases and control groups.

### 3.2. Health-Related Effects and Exposure to Inorganic Fertilizers

Forty-six studies (321 associations) assessed inorganic fertilizers in general (50%), nitrogen fertilizers (21%), phosphorus fertilizers (26%), and potassium fertilizers (3%). Nearly 25% (fifteen) of the studies considered multiple time periods for exposure, evaluating mostly pediatric malignancies. Occupational exposure was assessed in 87% of the total associations; in 4%, the exposure was reported to be jointly examined with other chemicals, and in 0.6% with the concomitant use of pesticides. Twelve studies (114 associations) pertained to exposure during the production of fertilizers or exposure via residential proximity to fertilizer plants, which is expected to be long-term and high for exposure during production and low or moderate for residential exposure. The rest of the studies referred to exposure to inorganic fertilizers during application or in residential vicinity to fields where fertilizers were applied, an exposure considered intermittent and low or moderate.

Most of the proposed associations pertained to solid organ tumors and hematologic malignancies (47%), followed by general symptoms, such as skin lesions, neurological (15%), respiratory (32%), and other diseases. The remaining associations, with under three studies per outcome, pertained to congenital disorders, developmental and hematological diseases, infections, rheumatic diseases, diabetes, mortality, neurological/vascular diseases, and psychiatric diseases.

#### 3.2.1. Neurological Outcomes

We found three case-control studies assessing exposure to inorganic fertilizers and neurological diseases in the USA. The major study limitations include the lack of replication, the small sample size, and residual confounding.

Yu et al. [71] explored the role of environmental factors in the development of amyotrophic lateral sclerosis (ALS, n = 66). For an exposure period ranging from 10 to 30 years, self-reported use of fertilizers to treat private yards and gardens was significantly associated with ALS (OR = 2.97, 95% CI 1.01 to 8.76).

Ton et al. [63] evaluated the risk of narcolepsy due to occupational and non–occupational factors, including fertilizers; they found a significant increase in narcolepsy for the highest levels of exposure to fertilizers (ncases = 67, ncontrols = 95; OR = 3.1, 95% CI 1.1 to 9.1).

Exposure to fertilizers, concomitantly with pesticides, has been significantly associated with an increased risk of developing vascular dementia. According to the Canadian Study of Health and Aging, individuals with occupational exposure to pesticides and fertilizers had more than twofold increased risk for vascular dementia compared with those without such exposure (OR = 2.60, 95% CI 1.30 to 5.23) [45].

#### 3.2.2. Other Non-Malignant Outcomes

In our systematic review, we identified numerous studies related to non-malignant outcomes that do not constitute discrete clinical entities; these findings are detailed in Supplementary Tables S2 and S3, provided in the Supplementary materials.

The association of congenital disorders with a variety of exposures, including inorganic fertilizers, was evaluated in the study conducted by Kristensen et al. [44]. A total of 4565 cases of congenital disorders among 192,417 farmers’ births were identified through the linkage of the Medical Birth Registry of Norway to data available from five agricultural and horticultural censuses (1969–1989). Polydactyly was associated with areas where over 25 kg/hectare of phosphorus fertilizer was used (adjusted OR = 1.85, 95% CI 1.15 to 2.99). Syndactyly was associated with a high nitrogen/phosphorus fertilizer ratio (adjusted OR = 1.6, 95% CI 1.04 to 2.46).

Parks et al. [53] explored how using fertilizers alongside pesticides or other agricultural exposures affects rheumatoid arthritis development among spouses of licensed pesticide applicators in the Agricultural Health Study. From 1993 to 2010, among 275 cases and 24,018 non-cases, a significant association was found with inorganic fertilizer application (OR = 1.7, 95% CI 1.1 to 2.7). After excluding cases diagnosed in the first two years of follow-up, the association was not statistically significant (OR = 1.4, 95% CI 0.8 to 2.6).

In a study conducted on workers at a phosphate fertilizer production facility, the mortality because of all causes was slightly elevated (standardized mortality ratio (SMR) = 1.07, 95% CI 1.02 to 1.13), indicating a relatively modest increase in all-cause mortality among the exposed workers compared with the general U.S. population [70].

#### 3.2.3. Cancerous Outcomes

Associations between inorganic fertilizers and cancerous outcomes were reported in 27 studies, which reflected 146 associations in our database. The list of cancer-related outcomes was heterogeneous, even for outcomes referring to the same clinical condition, making it difficult to find studies with a harmonized outcome definition.

##### Solid Organ Tumors

The association between exposure to inorganic fertilizers and solid-organ tumors was investigated in 17 studies (89 associations). These studies referred to recruitment periods ranging from 1945 to 2006, with most conducted in Europe [17,22,24,26,27,43,48,50,51,54,59] and the USA [18,22,32,34,60,70] and only one in Russia [13]. Regarding the study design, ten used a case-control design, four were exposure cohorts, and one study each used a cross-sectional and ecological design. The studies mostly assessed exposure to inorganic fertilizers during adulthood and, to a lesser extent, during childhood, infancy, or preconception. Gastric cancer, represented by five studies [13,17,18,24,70], and Central Nervous System (CNS) tumors, represented by five studies [22,50,51,60,70], are the cancer types more extensively assessed. Studies reporting on overall cancer incidence are reported in detail below.

In a historical cohort study conducted by Fandrem et al. (1993) [24], the incidence of cancer among 2023 male workers at a Norwegian fertilizer plant who were exposed to nitrate dust from 1945 to 1979 was tracked from 1953 to 1988 and compared with national cancer rates. No significant associations were found between cumulative nitrate exposure, employment duration, and the incidence of gastric cancer or any of the other nine cancers studied.

Bulbulyan et al. [13], in a cohort of Russian workers involved in fertilizer production and other services from 1965 to 1990, examined the impact of carcinogenic N-nitroso compound precursors on cancer-related mortality, including gastric cancer, using an industrial hygiene survey. These workers, recruited between 1945 and 1985, did not show higher mortality rates from all causes or neoplasms compared with the residents of the Moscow region. However, males with high cumulative exposure to nitrogen oxides (>40 unit-years) showed a twofold increase in gastric cancer mortality, and a marginally significant trend was shown for both genders. Additionally, significant arsenic exposure, a byproduct of sulfur dioxide production, correlated with higher overall and gastric cancer mortality. Notably, male workers experienced a significantly increased standardized mortality ratio for all cancers (SMR = 143, 95% CI 103–192) and lung cancer (SMR = 186, 95% CI 108–297) after a latency of over 20 years.

Similarly, Yiin et al. [70] estimated mortality trends among 3,199 workers employed between 1951 and 1976 at a phosphate fertilizer production facility in central Florida and followed up through 2011. Mortality due to stomach cancer was not statistically associated with occupation at the phosphate fertilizer plant (SMR = 0.91, 95% CI 0.44 to 1.68). All-cause mortality (SMR = 1.07, 95% CI 1.02 to 1.13), any cancer–related mortality (SMR = 1.16, 95% CI 1.06 to 1.28), lung cancer mortality (SMR = 1.32, 95% CI 1.13 to 1.53) and leukemia mortality (SMR = 1.74, 95% CI 1.11 to 2.62) were all statistically significantly increased compared with the general U.S. population as reference.

Two case-control studies by Cocco et al. investigated the link between gastric cancer and inorganic fertilizer exposure through farming. The first study [17] involved 640 cases and 959 controls in Italy, utilizing a job-exposure matrix, and found no significant association for individuals with over 21 years of exposure. The second study [18] analyzed 41,957 gastric cancer fatalities across 24 U.S. states from 1984 to 1996, also using a job-exposure matrix, and similarly found no link between inorganic fertilizer exposure and gastric cancer mortality.

A study by Musicco et al. examined the relationship between farmers’ occupational exposure to fertilizers and the development of gliomas [50]. The analysis revealed that farmers engaged in agricultural activities exclusively after 1960 had a significantly increased risk of developing gliomas (RR = 5.7, *p* = 0.028). Additionally, this study noted that the relative risk for farmers who worked both before and after 1960 was 2.5, although this was not statistically significant (*p* = 0.171). These findings suggest a potential link between the use of modern agricultural chemicals, including fertilizers, and an increased risk of gliomas, particularly in those with prolonged or exclusive exposure after the widespread adoption of these chemicals. Another study by Musicco et al. investigated the risk of gliomas among individuals with occupational exposure to agricultural chemicals, including fertilizers [51]. The results indicated that farmers exposed to fertilizers had a non-statistically significant risk of developing gliomas (RR = 1.4, 95% CI 0.8 to 2.49). However, when considering combined exposure to fertilizers, herbicides, insecticides, and fungicides, the relative risk was statistically significant (RR = 1.6, 95% CI 1.04 to 2.53). This highlights that combined exposure to multiple agricultural chemicals may pose a more substantial risk.

A study by Yiin et al. also investigated mortality among workers at a phosphate fertilizer plant, with a focus on CNS tumors, among other causes [70]. The standardized mortality ratio for CNS tumors was 0.97 (95% CI 0.44 to 1.83), indicating no significant increase in CNS tumor mortality compared with the general U.S. population.

Two studies examined the potential association between exposure to fertilizers and CNS tumors in children. The first study by Efird et al. investigated the relationship between maternal exposure to fertilizers and the subsequent development of CNS tumors in offspring [22]. The analysis revealed a statistically significant association (OR = 1.8, 95% CI: 1.1–3.0), indicating that maternal exposure to fertilizers during the five years prior to childbirth may contribute to an elevated risk of CNS tumors in children. The second study by Schwartzbaum et al. investigated the association between exposure to fertilizers and the risk of developing various childhood cancers, including neuroblastoma [60]. The results showed that parental gardening with fertilizers, herbicides, and pesticides was associated with neuroblastoma, although this association was not statistically significant and might be influenced by factors such as confounding and chance (OR = 1.1, *p* = 0.78).

##### Blood-Related Malignancies

Four studies [14,25,49,70] (one exposure cohort, two case-control, and one ecological study) assessed the association between exposure to inorganic fertilizers and multiple myeloma. Moreover, eight studies [12,25,40,60,61,68,69,70] examined exposure to fertilizers and the development of leukemia (four for acute, two for chronic, and two for leukemia in general), while three studies [25,60,69,70] evaluated the association between exposure to fertilizers and the development of lymphoma.

Yiin et al. [70] reported increased mortality due to multiple myeloma among workers in a phosphorus fertilizer plant compared with the general US population (ncases = 6, SMR = 3.01, 95% CI 1.1 to 6.55), a result based on only six cases.

In the multicenter population-based case-control study conducted by Morris et al. [49], 698 individuals with newly diagnosed multiple myeloma and 1683 controls were interviewed about the use of a variety of chemicals, including fertilizers. There was no statistically significant association.

Cantor et al. [14], in their case-control study, evaluated the risk of death due to multiple myeloma among males from 411 death certificates for the period 1968–1976 in the state of Wisconsin. They found a marginally statistically significant association between farmers who were fertilizer applicants in the highest intensity county strata compared with non-farmers who lived in the counties with the lowest intensity (OR = 1.7, 95% CI 1.0 to 2.9).

Fluegge et al. [25] analyzed data from the Healthcare Cost and Utilization Project to explore the link between nitrogen fertilizer use on farms and hospitalizations for blood-related cancers. Using Poisson regression while adjusting for non-farm use of nitrogen fertilizers and pesticides, they found a significant inverse association for multiple myeloma (IRR = 0.92, 95% CI 0.86 to 0.99). The adjusting covariates included not only the use of other pesticides and fertilizers but also socio-demographic factors, such as age, sex, and urbanization level, and environmental exposures, such as nitrous oxide (N_2_O) emissions.

Two hospital-based case-control studies conducted by Wong et al. examined a population of 722 confirmed acute myeloid leukemia (AML) cases and 1444 individually gender-age-matched controls in Shanghai, China [68,69], aiming to investigate the relationship between environmental exposures, including the use of fertilizers, and the risk of developing specific subtypes of AML. The findings revealed a statistically significant association between fertilizer exposure and risk of AML overall (OR = 1.53, 95% CI 1.16 to 2.04). When analyzing specific subtypes, the study reported an elevated but not statistically significant risk for acute promyelocytic leukemia (APL), characterized by the translocation t (15; 17) (OR = 1.62, 95% CI 0.63 to 4.10). Furthermore, the risk associated with AML with multilineage dysplasia (AML-MD), a subtype involving dysplasia in multiple cell lineages, was found to be significantly higher (OR = 1.89, 95% CI 1.26 to 2.82). The relationship between exposure to fertilizers and the development of acute leukemia, particularly in children, has been explored in a few studies. An exploratory case-control study by Shi et al. [61], including 201 new cases of childhood acute leukemia in Shanghai, found a statistically significant association between paternal exposure to chemical fertilizers within three months before pregnancy and the risk of childhood acute leukemia (OR = 9.5, 95% CI 1.1 to 79.6). A study by Schwartzbaum et al. [60] investigated environmental factors related to childhood cancers and identified that parental gardening with fertilizers, herbicides, and pesticides during the postnatal period was associated with a weak potential risk of developing various childhood cancers, including an elevated risk for acute lymphocytic leukemia (ALL) (OR = 1.3, 95% CI not reported). However, because the 95% confidence interval for this odds ratio was not reported, it is difficult to assess the precision and statistical significance of this potential risk.

As for lymphoma, the aforementioned study by Fluegge et al. [25] explored the relationship between nitrogen fertilizer use and the risk of blood-related cancers, including lymphoma. The results indicated that increased farm use of nitrogen fertilizers was associated with a reduced incidence rate ratio (IRR) for non-Hodgkin’s lymphoma (NHL) (IRR = 0.92, 95% CI 0.87 to 0.98), suggesting a potential inverse effect of nitrogen fertilizers against hospitalization for NHL. However, this finding also highlights the complexity of environmental exposures and the potential for confounding factors, such as nitrous oxide emissions, which may interact with other chemicals to influence cancer risk.

Schwartzbaum et al. [60], as we mentioned above, investigated the relationship between environmental exposures, including the use of fertilizers, herbicides, and pesticides in gardening, and the development of childhood cancers, such as non-Hodgkin’s lymphoma (NHL) and Hodgkin’s lymphoma (HL). The results showed that parental gardening with these chemicals during the postnatal period was weakly associated with NHL (OR = 1.3, 95% CI not reported) and HL (OR = 1.4, 95% CI not reported), indicating a possible association between these exposures and the risk of lymphoma. However, since the 95% confidence intervals for these odds ratios were not provided, the precision and statistical reliability of these associations remain unclear. Without this information, it is challenging to determine whether the observed associations are statistically significant or if they could be due to chance. Therefore, these findings should be interpreted with caution until more data are available to confirm the results.

A study by Yiin et al. [70] evaluated the mortality among workers at a phosphate fertilizer production facility, focusing on specific causes of death, including lymphoma. The analysis did not find any increase in mortality from non-Hodgkin’s or Hodgkin’s lymphoma.

### 3.3. Health-Related Effects and Exposure to Organic Fertilizers

Nineteen studies evaluated the use of organic fertilizers, pertaining to ninety-three associations. Various types of organic fertilizers were studied: sewage sludge (40%), animal manure (14%), dairy/veal manure (11%), swine manure (11%), human excreta (13%), organic fertilizer in general (6%), animal fertilizer or reuse of animal fertilizer (3%), fertilizer containing hooves and horn (1%), and natural fertilizers (1%). Exposure to organic fertilizers was not consistently and adequately described by the eligible studies, and therefore, distinct groups of studies used a different definition of this type of fertilizer. With regards to the endpoints under study, infections and symptom-related outcomes were most frequently assessed, represented by 44% of the associations each. The remaining outcomes related to allergy (5%), rheumatic (3%), or oncological, hematological, and respiratory diseases (each represented by only one study). Almost 75% of the studies included participants who were occupationally exposed to organic fertilizers, and 5 out of 19 studies examined exposure to organic fertilizers during certain lifetime windows. As with inorganic fertilizers, these five studies mostly pertained to single studies with poor replication validity.

Table 3 presents studies that explore the association between exposure to organic fertilizers and various health outcomes they address.

#### 3.3.1. Infectious-Related Outcomes

We found 11 studies, 2 case-control, 6 cross-sectional, 1 ecological study, and 2 nested case-control studies, estimating exposure to organic fertilizers and infectious endpoints, pertaining to 12 different infection-related outcomes and 35 associations. The reported outcomes were schistosomiasis, community- and hospital-acquired methicillin-resistant *Staphylococcus aureus* (MRSA), skin/soft tissue infection, Q fever-seroprevalence, Creutzfeldt–Jakob disease, cutaneous leishmaniasis, *Escherichia coli* O157:H7 antibodies, helminth infection, household poultry testing positive for *Campylobacter jejuni* (*C. jejuni*), malaria and soil-transmitted helminth.

Casey et al. [16], in a nested case-control study within a Pennsylvania health care system (2005–2010), examined the association between exposure to industrial swine and dairy/veal agriculture with the risk of MRSA and skin and soft-tissue infection (SSTI). Using electronic health records, they recruited primary care patients, classified them as either community-associated MRSA (n = 1539) or healthcare-associated MRSA (n = 1335), and then the cases were frequency-matched to controls (n = 2914) and patients with SSTI (n = 2895). Exposure was assessed via seasonal crop field manure application, as well as the number of livestock animals at the operation. Statistically significant odds ratios were reported for the highest quartile of swine manure exposure (community-associated MRSA, adjusted OR = 1.38, 95% CI, 1.13 to 1.69), healthcare-associated MRSA (adjusted OR = 1.30, 95% CI, 1.05 to 1.61), SSTI (adjusted OR = 1.37, 95% CI 1.18 to 1.60). The highest versus lowest quartile of dairy/veal was also associated with an increased risk of community-acquired MRSA infection (adjusted OR = 1.24, 95% CI 1.01 to 1.52).

A nested case-control study in Hanam, Vietnam, by Pham-Duc et al. [56] assessed the diarrhea risk in 464 adults exposed to wastewater and excreta. Statistically significant associations were found between diarrhea and composting of human excreta in households for less than three months (OR = 2.4, 95% CI 1.4 to 4.3), handling human excreta in fields (OR = 5.4, 95% CI 1.4 to 21.1), using animal excreta as fertilizer (OR = 1.6, 95% CI 1.0 to 2.6), and handling animal excreta in fields (OR = 3.3, 95% CI 1.8 to 6.0).

A recent outbreak of cutaneous Leishmaniasis (cL) by *Leishmania tropica* in Morocco led Gijon-Robles et al. [29] to determine its risk factors through a case-control study. Accumulation of organic fertilizers around homes was not related to the disease (OR = 2.31, 95% CI 0.51 to 10.4).

Van Duijn et al. [65], in a case-control study of 810 Europeans evaluating CJD, reported that exposure to fertilizers containing hooves and horns was significantly associated with the development of the disease (OR = 2.32, 95% CI 1.38 to 2.91).

A study conducted by Dal Pozzo et al. [19] in Southern Belgium assessed seroprevalence of *Coxiella burnetti* (*C. burnetti*), the cause of Q or Query fever, among veterinarians on potential risk factors of exposure to *C. burnetti*. Contact with manure within the last month was identified as a significant risk factor (OR = 6.77, 95% CI 1.8 to 25.45), while contact with manure 1 to 6 months before the study was not (OR = 1.67, 95% CI 0.28 to 10.09).

Carlton et al. [15] investigated two rural counties in Sichuan, China, where Schistosomiasis by *Schistosoma japonicum* had re-emerged despite control efforts. They evaluated the association between using human excreta as a fertilizer, also called “night soil”, and infection rates. In 2007, a total of 2005 participants were screened for Schistosoma infection, followed by 1365 participants in 2010, while the head of each household was interviewed about their agricultural practices. Nearly half of the households reported use of night soil as a fertilizer, both in 2007 (56%) and 2010 (46%). Statistically significant associations were also found between night soil use and infection caused by *S. japonicum* in 2007. It was shown that low-, medium-, and high-volume night soil use was significantly associated with schistosomiasis for participants recruited during 2007, showing increasing risks of schistosomiasis with higher volumes of night soil used (aOR = 5.67, 95% CI 1.98 to 16.22, aOR = 8.5, 95% CI 2.85 to 25.35 and aOR = 10.80, 95% CI 3.25 to 35.87, respectively).

Four [11,23,28,57] cross-sectional studies and an ecological study [73] examined infectious outcomes, such as infection of *C. jejuni* and *Escherichia coli* O157:H7, Malaria, and Soil-Transmitted Helminthiasis (STH), which were associated with exposure to organic fertilizers, with four 11,23,28,73 yielding statistically significant results.

#### 3.3.2. Allergies and Atopy

Illi et al. [37], in a cohort study in rural regions of Austria, Germany, and Switzerland, recruited 79,888 school-aged children to investigate the effects of farm exposures on asthma and atopy. Parents answered detailed questionnaires about distinct farm exposures in wave I, and in wave II, stratified random samples of 8419 children were taken, involving blood samples, genetic analyses, dust sampling, and specific IgE levels were available for 7682 children. Early-life farm exposure to manuring, especially during pregnancy and early childhood was associated with lower risk of asthma (aOR = 0.65, 95% CI 0.47 to 0.9), hay fever (aOR = 0.51, 95% CI 0.33 to 0.8), and atopic dermatitis (aOR = 0.66, 95% CI 0.45 to 0.96), but not for atopic sensitization (aOR = 0.85, 95% CI 0.65 to 1.11). The associations were adjusted for several confounders, but when authors performed multivariate stepwise weighted regression models that included contact with cows and with straw, the protective effect of manuring was not statistically significant. Also, maternal involvement in manuring during pregnancy was significantly associated with a decreased risk of atopic dermatitis in their offspring.

#### 3.3.3. Other Non-Malignant Outcomes

Parks et al. [53] investigated the association of concomitant use of fertilizers with pesticides or other agricultural exposures with rheumatoid arthritis and found that the application of natural fertilizers was not associated with rheumatoid arthritis (OR = 0.85, 95% CI 0.48 to 1.5). Issaragrisil et al. [38] performed a case-control study in Thailand, enrolling 541 patients and 2261 controls from 1989 to 2002, to investigate the association of aplastic anemia with several occupational parameters. The use of animal fertilizers showed a marginally statistically significant increased risk with the development of aplastic anemia (RR = 2.1, 95% CI 1.0 to 4.4).

#### 3.3.4. Cancerous Outcomes

The only case-control study by Menvielle et al. [48] evaluating various occupational exposures as risk factors for lung cancer in New Caledonia (n = 533, 10 exposed) failed to observe an association.

## 4. Discussion

In this systematic review, we appraised 65 studies that involved 349,033 participants, assessing 407 associations related to exposure to inorganic and organic fertilizers. Based on the comprehensive evaluation of the available evidence, we found various single statistically significant associations between exposure to inorganic fertilizers and outcomes relevant to multiple myeloma, leukemia, and lymphoma, as well as between exposure to organic fertilizers and diarrhea and infections. However, these findings should be interpreted with caution, given the variability in study quality and design. No robust associations were observed between solid organ tumor development and any fertilizer type. Our evidence synthesis investigated several approaches to address exposure to fertilizers, considering both environmental and occupational contact with inorganic and organic types. We explored different life stages (preconception through adulthood) to evaluate whether fertilizers have major health outcomes. A quantitative synthesis was not deemed appropriate due to the observed heterogeneity in the exposure assessment, exposure windows, outcome definition, and population characteristics.

There is a suggestion that carcinogenesis from fertilizers is caused by nitrate in drinking water, elevated because of nitrogen fertilizer use [74,75,76,77,78,79,80,81,82]. This hypothesis is supported by mechanistic data, but the relevant epidemiological evidence is characterized by methodological limitations. When nitrogen fertilizers are applied to crops, they can leach into groundwater and surface water, leading to elevated nitrate concentrations [76]. When digested through water consumption, nitrate is metabolized into nitrite by bacteria present in the oral cavity and gut. This conversion is a critical step because nitrite is more chemically reactive than nitrate. In the acidic environment of the stomach, nitrite can react with secondary amines and amides, which are commonly found in foods, to form N-nitroso compounds (NOCs). These NOCs are recognized as powerful carcinogens in animal studies and are believed to have similar cancer-causing effects in humans [81]. The Pelayo Correa’s study [78] was among the first to propose that dietary exposure to nitrates and nitrites could increase the risk of gastric cancer by promoting the formation of N-nitroso compounds in the stomach, particularly under conditions of chronic atrophic gastritis and under the interplay of various other factors, including dietary components, such as antioxidants. This model laid the foundation for future research on the role of diet and environmental factors in gastric cancer development. For example, Bulbulyan et al. [13] indicate an elevated mortality risk for gastric and lung cancer among male workers with high cumulative exposure to nitrogen oxides and arsenic. Although the potential confounding effect of smoking, a known risk factor for both gastric and lung cancers, cannot be dismissed due to the absence of specific smoking status data within the cohort, the evidence suggests that smoking alone is unlikely to account for the observed excess risk of lung cancer, a notion supported by the lack of increased mortality from other smoking-related diseases, including circulatory diseases and various cancers. The underlying mechanism of the remaining proposed effects in the body of evidence under study remains unclear. Kristensen et al. [44] found a significant association between polydactyly in areas with high phosphorus fertilizer use, as well as between syndactyly and a high Nitrogen/Phosphorus fertilizer ratio in Norwegian farmers’ births. These single-study findings are important in terms of hypothesis formulation; however, they were reported in one study of Norwegian farmers only and may be influenced by genetic factors, as well as other environmental exposures, such as pesticides and herbicides, and other factors, such as nutritional deficiencies, socioeconomic status.

The findings from this synthesis effort suggest a possible link between organic fertilizers and infection-related outcomes. Organic fertilizers made from materials such as livestock manures, compost, vermicompost, and sewage sludge vary in infectious disease risk according to the origins and proportions of the ingredients [2,83]. Microorganisms in organic fertilizers, such as bacteria and fungi, threaten plant and human health if not fully broken down. Van Duijn et al. [65] found a statistically significant association between exposure to organic fertilizers and the risk of Creutzfeldt–Jakob disease (CJD). This suggests that environmental exposures, such as fertilizer use, may play a role in the transmission of prion diseases. Additionally, previous studies have suggested that CJD transmission could occur through the inhalation of meat aerosols [84,85,86]. Furthermore, prion proteins have been detected in feces, indicating potential fecal–oral transmission routes [87]. These findings highlight the need to consider multiple pathways, including both environmental exposures and biological transmission routes, in understanding and mitigating prion disease transmission. In addition, the storage and maintenance state of organic fertilizers affects the risk of infectious diseases. Improper procedures during composting can lead to the development of pathogenic bacteria, fungi, and Actinomycetes, turning organic matter into breeding sites for disease-carrying flies and mosquitoes. Runoff from fields where organic fertilizers were used can pollute water springs with pathogens. The risk of infection is also linked to hygiene practices in agricultural settings, where inadequate measures lead to an increased risk of pathogen exposure. Studies associate organic fertilizers with microorganisms and antimicrobial resistance (AMR) [88]. AMR, an evolutionary adaptation of microbes, can be intrinsic or acquired through gene mutations or assimilation of DNA (in bacteria, horizontal transfer of mobile genetic elements takes place [89,90]), frequently from untreated human and animal excreta or antimicrobial manufacturing, which are the leading causes of widespread environmental release of biological antimicrobial resistant pollutants [91,92,93]. Manure, a reservoir of antibiotic-resistant genes, can introduce antibiotic-resistant bacteria and residues into the environment, changing microbial populations. In China, an eightfold higher absolute copy number of antibiotic-resistant genes has been reported in manure-fertilized organic lettuce compared with the conventionally produced [94]. Common pathogens detected in soil are extended-spectrum β-lactamase-producing Enterobacteriaceae (ESBL-Ent), methicillin-resistant *Staphylococcus aureus* (MRSA) and vancomycin-resistant enterococci (VRE) [95], thus manure use in ready-to-eat crops is discouraged in some European countries (e.g., UK and Germany).

Pesticide and fertilizer co-exposure (often used together) can introduce complexities and potentially skew results, affecting the validity of the evidence. This may lead to inaccurate conclusions about the association between fertilizer exposure and health outcomes. Moreover, investigating dose–response relationships for fertilizers and pesticides is challenging because of complicated interactions, hindering the ability to show clear dose–response patterns. In addition, the combined effects of pesticides and fertilizers may over/underestimate risks, making it harder to assess total exposure and understand true risks. As previously stated, the most used study design in our systematic review was case-control studies, which provided limited information about exposure assessment. While case-control studies can reconstruct the occupational history of participants, cohort studies often capture exposure information only at the time of recruitment. To better capture the complexity of co-exposure, longitudinal cohort studies that follow individuals at multiple stages over time can be considered a better study design. This approach allows for the collection of continuous exposure data to the same types of fertilizers and co-exposures, providing a more comprehensive understanding of the risks. Inorganic fertilizers contain higher concentrations of heavy metals than organic ones, leading to soil heavy metal pollution [96] with toxic elements, such as As, Cd, and Pb [97]. Chronic kidney disease of unknown etiology (CKDu) in Sri Lanka has been traditionally associated with Cd levels in food and soil samples [98,99,100]. Although high concentrations of cadmium could originate from the use of phosphate fertilizers [39,101,102,103,104], other researchers argue that elevated cadmium levels in the soil are attributed to natural geographical sources and environmental factors, such as acid rain from coal-fired power stations [105].

Potential limitations to our work include a significant number of studies of a retrospective design, thus making it more prone to recall bias. The assessment of exposure to fertilizers was problematic, with some studies using objective measures of exposure while others defined exposure in a more subjective way. Furthermore, co-examination of fertilizers and pesticides made it difficult to discern their effects. Additionally, population heterogeneity, exposure definition variability, outcome diversity, and restricted data availability in the field made meta-analysis impossible to conduct. Differences in fertilizer use across high-, medium-, and low-income countries, such as higher chemical application rates in higher-income nations versus lower rates in lower-income ones [106,107], along with precautionary measures during handling and the under-representation of continents, such as Latin America, increase heterogeneity for further analyses. Additionally, studies assessing specific exposure windows (e.g., preconception or pregnancy) with retrospective or cross-sectional designs may not adequately address other confounders and are likely to suffer from replication validity issues. Another potential limitation is the high proportion of descriptive studies included in our work, as these types of studies are not capable of proving causal relationships.

To our knowledge, this is the first synthesis effort describing the effect of fertilizers as a distinct exposure entity in human health. We believe that this information is valuable for authorities and regulatory agencies in developing public health policies and interventions, minimizing unnecessary exposure to fertilizers, and guiding farmers on protective equipment. Given the existing disparities in available studies, we advocate for the development of an international research protocol to systematically address inherent limitations, foster result harmonization, and establish comparability and reproducibility across studies. As precision agriculture and sustainable practices continue to evolve, farmers and researchers must work together to maximize the benefits of fertilizers while minimizing their environmental footprint and associated challenges.

## Figures and Tables

**Table 1:** PECO Statement of the systematic review.

Element	Description
Population	Humans of all age groups without language or geographical limitations
Exposure	Exposure to fertilizers (inorganic and organic) through occupational or residential exposure
Comparator	Non-exposed individuals or those with lower levels of exposure
Outcome	Health-related outcomes including cancer, multiple myeloma, infections, diarrhea, amyotrophic lateral sclerosis, rheumatoid arthritis, narcolepsy, polydactyly, aplastic anemia, and Creutzfeldt–Jakob disease

**Table 2:** Studies associated with exposure to inorganic fertilizers and health outcomes.

Health Outcome	Type of Exposure	Study Type	Results	Quality Assessment
**Non-Malignant Outcomes**				
**(a) Infectious Diseases**				
**Analytical Studies**				
Creutzfeldt–Jakob disease (CJD)	Both	Case-control	Inverse association, NS	Good
Tuberculosis	Occupational	Exposure Cohort	Inverse association, NS	Poor
**(b) Allergies and Atopy**				
**Descriptive Studies**				
Asthma	Both	Cross-sectional	Adverse association, NS	Moderate
Asthma	Occupational	Cross-sectional	Adverse association, NS	Moderate
**(c) Neurological Outcomes**				
**Analytical Studies**				
Amyotrophic Lateral Sclerosis (ALS)	Both	Case-control	Adverse association, NS	Moderate
Narcolepsy	Occupational	Case-control	Adverse association, NS	Moderate
Vascular Dementia	Occupational	Case-control	Adverse association *	Moderate
**(d) Other Outcomes**				
**Analytical Studies**				
Alcoholism	Occupational	Cohort	Inverse association, NS	Poor
All-cause mortality	Occupational	Cohort	Adverse association *	Poor
Aplastic Anemia	Both	Case-control	Adverse association, SNR	Moderate
Esophageal atresia	Occupational	Cohort	Adverse association, NS	Moderate
Polydactyly	Occupational	Cohort	Adverse association, NS	Moderate
Rheumatoid Arthritis	Occupational	Cohort	Adverse association *	Moderate
Skin conditions (rash/lesions/ulcer)	Occupational	Case-control	Adverse association, NS	Moderate
Skin conditions (rash/lesions/ulcer)	Occupational	Cohort	Adverse association **	Poor
Syndactyly	Occupational	Cohort	Adverse association *	Moderate
**Descriptive Studies**				
Bronchitis (acute and chronic)	Environmental	Cross-sectional	Inverse association, NS	Moderate
Bronchitis (acute and chronic)	Environmental	Cross-sectional	Adverse association, NS	Moderate
Bronchitis (acute and chronic)	Environmental	Cross-sectional	Adverse association *	Poor
Bronchitis (acute and chronic)	Occupational	Cross-sectional	Adverse association *	Moderate
Emphysema	Environmental	Cross-sectional	Adverse association *	Poor
Skin conditions (rash/lesions/ulcer)	Environmental	Cross-sectional	Adverse association *	Poor
**Malignant Outcomes**				
**Analytical Studies**				
Acute Leukemia (AML, ALL, ANLL)	Occupational	Case-control	Adverse association *	Moderate
Acute Leukemia (AML, ALL, ANLL)	Occupational	Case-control	Adverse association *	Poor
Acute Leukemia (AML, ALL, ANLL)	Occupational	Case-control	Adverse association *	Moderate
All Cancer Mortality	Occupational	Exposure Cohort	Adverse association, NS	Poor
All Cancer Mortality	Occupational	Exposure Cohort	Adverse association *	Poor
All Cancer Mortality	Occupational	Exposure Cohort	Inverse association, NS	Poor
B-cell neoplasms	Occupational	Case-control	Adverse association *	Moderate
Brain tumors (glioma, meningioma, neuroblastoma)	Occupational	Case-control	Adverse association, NS	Moderate
Brain tumors (glioma, meningioma, neuroblastoma)	Occupational	Case-control	Adverse association *	Moderate
Brain tumors (glioma, meningioma, neuroblastoma)	Occupational	Case-control	Adverse association *	Poor
Breast cancer	Occupational	Exposure Cohort	Inverse association, NS	Poor
Buccal cavity and pharynx Cancer	Occupational	Exposure Cohort	Inverse association, NS	Poor
Cancer of male genital organs	Occupational	Case-control	Adverse association *	Moderate
Cancer of male genital organs	Occupational	Exposure Cohort	Adverse association *	Moderate
Cancer of male genital organs	Occupational	Exposure Cohort	Adverse association, NS	Poor
Cancer of other male genital organs	Occupational	Exposure Cohort	Adverse association *	Poor
Cancer of other and unspecified organs	Occupational	Exposure Cohort	Adverse association, NS	Poor
Cancer of trachea, bronchus, and lung	Occupational	Exposure Cohort	Adverse association *	Poor
Chronic Leukemia (CLL, CML, SLL)	Both	Case-control	Adverse association *	Moderate
Chronic Leukemia (CLL, CML, SLL)	Occupational	Case-control	Adverse association, NS	Moderate
Colon cancer	Occupational	Exposure Cohort	Inverse association, NS	Poor
Connective tissue cancer	Occupational	Exposure Cohort	Inverse association, NS	Poor
Digestive system and peritoneum cancer	Occupational	Exposure Cohort	Inverse association, NS	Poor
Gastric cancer	Occupational	Case-control	Adverse association, NS	Moderate
Gastric cancer	Occupational	Case-control	Adverse Association, NS	Moderate
Gastric cancer	Occupational	Exposure Cohort	Inverse association, NS	Poor
Gastric cancer	Occupational	Exposure Cohort	Inverse association, NS	Poor
Gastric cancer	Occupational	Exposure Cohort	Adverse Association, NS	Poor
Germ cell cancer	Occupational	Case-control	Adverse Association, NS	Moderate
Hematological Malignancies	Occupational	Case-control	Adverse association *	Poor
Leukemia	Occupational	Exposure Cohort	Adverse association *	Poor
Lung cancer	Occupational	Case-control	Adverse association, NS	Moderate
Lung cancer	Occupational	Exposure Cohort	Adverse Association, NS	Poor
Lung cancer	Occupational	Exposure Cohort	Adverse association, NS	Poor
Monoclonal gammopathy of undetermined significance (MGUS)	Occupational	Case-control	Adverse association *	Poor
Monoclonal gammopathy of undetermined significance (MGUS)	Occupational	Case-control	Adverse association *	Moderate
Multiple myeloma	Both	Case-control	Adverse association, NS	Moderate
Myelodysplastic syndrome	Occupational	Case-control	Adverse association *	Good
Mesothelioma	Occupational	Exposure Cohort	Adverse Association, NS	Poor
Mesothelioma	Occupational	Exposure Cohort	Adverse Association, SNR	Poor
Pancreatic cancer	Occupational	Exposure Cohort	Adverse Association, NS	Poor
Pancreatic cancer	Occupational	Exposure Cohort	Adverse association, NS	Poor
Pharynx cancer	Occupational	Exposure Cohort	Inverse association, NS	Poor
Rectum cancer	Occupational	Exposure Cohort	Inverse association, NS	Poor
Rectum cancer	Occupational	Exposure Cohort	Inverse association, NS	Poor
Respiratory system cancer	Occupational	Exposure Cohort	Adverse association *	Poor
Skin cancer	Occupational	Exposure Cohort	Inverse association, NS	Poor
Skin cancer	Occupational	Exposure Cohort	Adverse association, NS	Poor
T/NK-cell neoplasms	Occupational	Case-control	Inverse association, NS	Moderate
Tongue cancer	Occupational	Exposure Cohort	Inverse association, NS	Poor
Uveal Melanoma	Occupational	Case-control	Adverse association *	Poor
Urinary tract cancer (Renal and bladder)	Environmental	Case-control	Adverse association, NS	Poor
Urinary tract cancer (Renal and bladder)	Occupational	Exposure Cohort	Adverse association, NS	Poor
Urinary tract cancer (Renal and bladder)	Occupational	Exposure Cohort	Adverse association, NS	Poor
**Descriptive Studies**				
Acute Leukemia (AML, ALL, ANLL)	Environmental	Cross-sectional	Adverse association, SNR	Moderate
Brain tumors (glioma, meningioma, neuroblastoma)	Environmental	Cross-sectional	Adverse association, SNR	Moderate
Ewing’s sarcoma	Environmental	Cross-sectional	Adverse association, SNR	Moderate
Leukemia	NR	Ecological study	Inverse association *	Poor
Leukemia	Occupational	Ecological study	Adverse association *	Moderate
Lymphoma (Hodgkin and Non-Hodgkin)	Environmental	Cross-sectional	Adverse association, SNR	Moderate
Multiple myeloma	NR	Ecological study	Adverse Association, NS	Poor
Multiple myeloma	Occupational	Ecological study	Adverse association *	Moderate
Non-Hodgkin Lymphoma	NR	Ecological study	Adverse Association, NS	Poor
Osteosarcoma	Environmental	Cross-sectional	Adverse association, SNR	Moderate
Ovarian cancer mortality	NR	Ecological study	Adverse association *	Poor
Wilm’s Tumor	Environmental	Cross-sectional	Inverse association, SNR	Moderate

Note: * *p* < 0.05, ** *p* < 0.01; NR: Not reported; NS: Not statistically significant; SNR: Significance not reported; Type of exposure: Occupational, Environmental, both; Quality Assessment: Good, Moderate, Poor.

**Table 3:** Studies associated with exposure to organic fertilizers and health outcomes.

Health Outcome	Type of Exposure	Study Type	Results	Quality Assessment
**Non-Malignant Outcomes**				
**(a) Infectious Diseases**				
**Analytical Studies**				
Community-acquired MRSA	Both	Nested case-control	Adverse association **	Moderate
Creutzfeldt–Jakob disease (CJD)	Both	Case-control	Adverse association **	Good
Cutaneous leishmaniasis	Environmental	Case-control	Adverse association (NS)	Poor
Hospital-acquired MRSA	Both	Nested case-control	Inverse association *	Moderate
Skin/soft tissue infection	Both	Nested case-control	Adverse association **	Moderate
**Descriptive Studies**				
Acute Bronchitis	Environmental	Cross-sectional	Inverse association **	Poor
*Escherichia coli* O157 antibodies	Environmental	Cross-sectional	Adverse association **	Good
Helminth Infection	Environmental	Cross-sectional	Adverse association (NS)	Poor
Household poultry positive for *C. jejuni*	Occupational	Cross-sectional	Adverse association **	Good
Malaria and Soil-Transmitted Helminthiasis	Environmental	Cross-sectional	Adverse association **	Moderate
Q fever seroprevalence against *C. burnetii*	NR	Cross-sectional	Adverse association **	Poor
Schistosomiasis	Environmental	Cross-sectional	Inverse association (NS)	Poor
**(b) Allergies and Atopy**				
**Analytical Studies**				
Asthma	Environmental	Cohort	Inverse association **	Good
Atopic dermatitis	Environmental	Cohort	Inverse association **	Good
Hay Fever	Environmental	Cohort	Inverse association **	Good
**(c) Other Outcomes, including symptoms**				
**Analytical Studies**				
Aplastic anemia	Occupational	Case-control	Adverse association **	Moderate
Rheumatoid Arthritis	Occupational	Cohort	Inverse association (NS)	Moderate
**Descriptive Studies**				
Chronic Bronchitis	Occupational	Cross-sectional	Adverse association **	Moderate
Emphysema	Environmental	Cross-sectional	Adverse association *	Poor
Skin ulcer	Environmental	Cross-sectional	Adverse association *	Poor
**Malignant Outcomes**				
**Analytical Studies**				
Lung cancer	Occupational	Case-control	Adverse association (NS)	Moderate

Note: * *p* < 0.05, ** *p* < 0.01; NR: Not reported; NS: Not statistically significant; Type of exposure: Occupational, Environmental, both; Quality Assessment: Good, Moderate, Poor.

## Data Availability

Our analysis is based on published data available in the literature, further information is given in the Section 2. The data extraction files are available upon request.

## Supplementary Materials

The following supporting information can be downloaded at: https://www.mdpi.com/article/10.3390/toxics12100694/s1, Supplementary Table S1: Quality assessment of the studies using RTI Item Bank; Supplementary Table S2: Studies associated with exposure to inorganic fertilizers and health outcomes; Supplementary Table S3: Studies associated with exposure to organic fertilizers and health outcomes; Supplementary Table S4: PRISMA-P Checklist. Reference [108] are cited in the Supplementary Materials.
