# Supplementary material for: Fertilizers and Human Health—A Systematic Review of the Epidemiological Evidence
Source: Toxics. 2024 Sep 26;12(10):694. doi: 10.3390/toxics12100694 (PMC11511508; doi:10.3390/toxics12100694)
Supplement: Supplementary file 1 [file toxics-12-00694-s001.zip › toxics-3021067-supplementary.pdf]

Supplementary Table S1. Quality assessment of the studies using RTI Item Bank.

PUBMED ID	Statistical method	Study design	Inclusion criteria clearly stated (yes, partially, no)	Authors mention power calculations	Level of detail in describing exposure (high, medium, low)	Robust measurement of exposure. (biomarker (yes); small area ecological measures, job titles, questionnai-re (partial); was based on large area ecological measures (no)	Were measures of exposure specific? yes; based on broader, chemically-related groups (partial); based on broad groupings of diverse chemical and toxicological properties (no)	Attempt to balance the allocation between the groups (e.g., through stratification, matching) in case-control studies	Adjustment for confounders (yes, some, no)	Assessors blinded to exposure status (for cohort studies)	Outcomes assessed using valid and reliable measures, implemented consistently across all study participants
3421243	Mantel's chi-square test	Retrospective	yes	no	low	partial	no	yes	yes	NA	yes
27285288	Logistic regression	Prospective	yes	no	medium	partial	no	-	yes	no	yes
27285288	Logistic regression models,	Prospective	yes	no	medium	partial	no	-	yes	no	yes
1951308	Mantel's chi-square test	Retrospective	no	no	low	partial	no	yes	no	NA	yes
3458965	Mantel's chi-square test	Retrospective	no	no	low	partial	no	yes	yes	NA	yes
19703505	Conditional logistic regression	Retrospective	yes	no	low	partial	no	yes	no	NA	yes
24979055	Conditional logistic regression	Retrospective	yes	no	low	partial	no	yes	no	NA	yes
24979055	Conditional logistic regression	Retrospective	yes	no	low	partial	no	yes	yes	NA	yes
12675788	Unconditional logistic regression	Retrospective	yes	no	high	partial	no	yes	yes	NA	yes
12883019	Unconditional logistic regression	Retrospective	yes	no	low	partial	no	yes	yes	NA	yes
22052342	Unconditional logistic regression	Retrospective	yes	no	low	partial	no	yes	yes	NA	yes
1990042	Conditional	Retrospectiv	yes	no	low	partial	no	yes	no	NA	yes

2	logistic regression	e									
2568087	Matched pairs analyses, McNemar's chi-square test with correction	Retrospective	yes	no	low	partial	no	yes	no	NA	yes
2568087	Logistic regression	Retrospective	yes	no	low	partial	no	yes	no	NA	yes
6582313	NR	Retrospective	yes	no	low	partial	no	yes	yes	NA	yes
6941037	McNemar test	Cross sectional	yes	no	low	partial	no	yes	no	NA	yes
8770459	Poisson regression	Prospective	yes	no	medium	partial	partial	n/a	yes	NR	yes
20519130	Unconditional logistic regression	Retrospective	yes	no	low	partial	no	yes	yes	NA	yes
23803538	NR	Retrospective	no	no	low	partial	no	yes	n/a	NA	yes
8061172	Logistic regression	Retrospective	yes	no	low	partial	no	yes	yes	NA	yes
17495700	Logistic regression	Cross sectional	yes	no	low	partial	no	n/a	yes	NA	yes
19900423	Conditional logistic regression	Retrospective	yes	no	low	partial	no	yes	no	NA	yes
9270956	Logistic regression	Prospective	yes	no	medium	partial	partial	n/a	yes	n/a	yes
9660576	Unconditional logistic regression	Retrospective	yes	no	low	partial	no	yes	yes	NA	no
20817628	Conditional logistic regression	Retrospective	yes	no	high	partial	no	yes	yes	NA	yes
8756092	Matched pairs analyses, McNemar's chi-square test with correction	Retrospective	yes	no	low	partial	no	yes	no	NA	yes

23194098	Logistic regression	Retrospective	yes	no	high	partial	no	no	yes	NA	yes
23194098	Logistic regression and Trend analysis with days of fertilizer use entered to logistic regression model as a continuous variable	Retrospective	yes	no	high	partial	no	no	yes	NA	yes
10658565	Logistic regression	Retrospective	yes	no	low	partial	no	yes	yes	NA	yes
30453431	Stratified multivariate logistic regression	Retrospective	no	no	high	partial	no	yes	yes	NA	yes
1311140	Unconditional logistic regression	Retrospective	yes	no	low	partial	no	yes	yes	NA	yes
10884442	Unconditional logistic regression	Prospective	yes	no	low	partial	no	n/a	yes	NA	yes
9056606	Unconditional logistic regression	Retrospective	yes	no	low	partial	no	yes	yes	NA	yes
12396522	Logistic regression	Cross sectional	yes	no	high	partial	no	n/a	yes	NA	no
12503714	Logistic regression	Cross sectional	yes	no	low	partial	no	n/a	yes	NA	no
22534534	Weighted logistic regression models using the Taylor series method	Cross sectional	yes	no	low	partial	ni	n/a	yes	n/a	yes
4065081	Regression	Prospective	yes	no	high	partial	partial	n/a	yes	yes	no
16155480	NR	Cross sectional	yes	no	low	partial	no	n/a	yes	n/a	no
9559405	Student's t-	Cross	yes	no	high	partial	partial	n/a	no	NA	yes

	test.	sectional									
1849220	Polytomous logistic regression	Cross sectional	yes	no	low	partial	no	n/a	yes	NA	yes
8905933	Logistic regression	Cross sectional	yes	no	medium	partial	partial	n/a	yes	NA	yes
29806200	NR	Retrospective	Yes	No	low	No	No	No	yes	NA	yes
28719770	NR	Cross sectional	Yes	no	low	partial	yes	No	yes	NA	yes
28709444	Poisson regression	Cross sectional	no	no	low	no	no	no	no	NA	NR
28709444	Poisson regression	Cross sectional	no	no	low	no	no	no	yes	NA	NR
26950029	NR	Retrospective	partially	No	low	no	no	yes	no	NA	yes
26749554	NR	Cross sectional	no	no	low	partial	partial	no	no	NA	yes
26566973	Univariate	Retrospective	partially	no	low	partial	no	yes	no	NA	yes
26566973	Logistic regression	Retrospective	partially	no	low	partial	no	yes	some	NA	yes
26046426	Bayesian-Poisson	Cross sectional	no	no	low	no	no	no	no	NA	yes
25596925	NR	Retrospective	no	no	low	no	no	no	no	NA	yes
25590142	Multi-level, fixed-effect logistic regression model	Prospective	no	no	medium	partial	partial	no	yes	no	yes
25590142	Multi-level, fixed-effect logistic regression model	Cross sectional	no	no	medium	partial	partial	no	yes	NA	yes
25239151	Logistic regression	Retrospective	partially	no	low	partial	partial	yes	no	NA	yes
24514575	Longitudinal linear mixed model		partially	no	low	partial	partial	no	some	NR	yes
24043228	Regression	Retrospective	yes	no	medium	partial	partial	yes	yes	NA	yes

23837685	Regression	Cross sectional	partially	no	low	partial	no	nr	no	NA	yes
23305757	Regression	Cross sectional	partially	yes	low	partial	partial	nr	some	NA	yes
21098630	Discrete time hazard model	Prospective	NR	No	low	partial	partial	NR	yes	NR	NR
16254144	Regression	Retrospective	yes	No	medium	partial	partial	yes	some	NA	yes
16254144	Regression	Retrospective	yes	No	medium	partial	partial	yes	no	NA	yes
12717628	Regression	Cross sectional	No	No	low	partial	partial	yes	some	NA	yes
11388516	Regression	Retrospective	yes	No	low	partial	partial	yes	some	NA	yes
11298587	Chi square	Retrospective	yes	yes	medium	partial	yes	yes	yes	NA	yes
25785490	Linear regression	Cross sectional	No	No	low	No	No	No	No	NA	No
24774694	Chi square	Cross sectional	yes	yes	medium	partial	No	No	No	NA	yes
23684270	Linear regression	Prospective	partially	no	high	yes	yes	NA	some	NR	yes
18171641	Cochran Armitage	Cross sectional	No	No	low	partial	No	NA	No	NA	No
17718171	Fisher's exact	Cross sectional	partially	No	high	partial	yes	No	some	NA	Yes
17718171	Paired samples t-test	Cross sectional	partially	No	high	partial	yes	No	some	NA	Yes
8685670	Poisson regression	Prospective	No	No	medium	partial	partial	No	some	NR	yes
7148804	NR	Retrospective	No	No	low	no	no	no	some	NA	yes
26523937	NIOSH Life Table Analysis System	Exposure cohort	yes	no	low	no	no	n/a	no	NA	yes
8393697	Poisson distribution	Exposure cohort	yes	no	low	no	no	no	no	NA	yes

Supplementary Table S2. Studies associated with exposure to inorganic fertilizers and health outcomes.

Health outcome	Number of studies for each health outcome	First author (year of publication)	Study type	Type of exposure (occupational / environmental / both)	Outcome metrics
Chest cold / illness / tightness	3	Kobrossi R. (2002)	Cross – sectional	Environmental	OR = 0.69 (0.25 – 1.9)
		Zhang LX (2002)	Cross sectional	Both	OR = 2.2 (1.6 – 2.9)
		Rahman (2007)	Cross sectional	Occupational	Chi^2, p = 0.02
Asthma	2	Zhang LX (2002)	Cross – sectional	Both	OR = 1.3 (0.9 - 1.8)
		Ballal SG. (1998)	Cross – sectional	Occupational	RR = 1.15 (0.62 – 2.15)
Bronchitis (acute & chronic)	4	Valcin M. (2007)	Cross – sectional	Environmental	OR = 0.84 (0.61 – 1.15) - chronic
		Kobrossi R. (2002)	Cross – sectional	Environmental	OR = 3.02 (0.76 – 12)
		Ballal SG. (1998)	Cross – sectional	Occupational	RR = 5.32 (1.72 – 16.08) - chronic
		Khuder (2007)	Cross – sectional	Environmental	Chi2, p = 0.042 - acute
Carbon monoxide diffusing capacity (DLco)	1	Hovland (2014)	Cohort	Occupational	Estimated change = -0.068 (-0.12, -0.019)
Lung function (Forced Expiratory Volume in 1 sec (FEV1), Forced Vital Capacity (FVC))	2	Hovland (2013)	Cohort	Occupational	-0.0031 (-0.014, 0.008) - FVC
		Rahman (2007)	Cross – sectional	Occupational	P = 0.05 (Not reported) – (FEV1)
Nasal symptoms	1	Gorman Ng M (2013)	Case – control	Occupational	OR = 2.02 (0.96 – 4.26)
Nosebleed	1	Gorman Ng M (2013)	Case – control	Occupational	OR = 2.18 (0.98 – 4.85)
Tuberculosis	1	Yiin JH (2016)	Exposure Cohort	Occupational	SMR = 0.71 (0.09 – 2.55)
Cough	5	Gorman Ng M (2013)	Case – control	Occupational	OR = 1.45 (0.67 – 3.15)
		Kobrossi R (2002)	Cross – sectional	Environmental	OR = 0.24 (0.07 – 0.89)<br><br>OR = 4.95 (1.36 – 17.9)- yearly episodes of increased cough and phlegm for 1 week or more
		Zhang LX (2002)	Cross – sectional	Both	OR = 1.3 (0.9 – 1.7)
		Ballal SG (1998)	Cross – sectional	Occupational	RR = 0.86 (0.48 – 1.52)
		Rahman (2007)	Cross – sectional	Occupational	Chi^2, p = 0.05
Phlegm	4	Gorman Ng M (2013)	Case – control	Occupational	OR = 0.73 (0.36 – 1.49)
		Kobrossi R (2002)	Cross – sectional	Environmental	OR = 1.89 (0.78 – 4.55)
		Zhang LX (2002)	Cross – sectional	Both	OR = 1.4 (1.1 – 1.9)

		Ballal SG (1998)	Cross – sectional	Occupational	RR = 0.79 (0.43 – 1.47)
Dyspnoea	1	Ballal SG (1998)	Cross – sectional	Occupational	RR = 1.13 (0.62 – 2.04)
Emphysema	1	Khuder (2007)	Cross – sectional	Environmental	Chi^2, p = 0.025
Wheezing	3	Kobrossi R (2002)	Cross – sectional	Environmental	OR = 2.56 (0.93 – 7.09)
		Zhang LX (2002)	Cross – sectional	Both	OR = 1.4 (1.1 – 1.8)
		Ballal SG (1998)	Cross – sectional	Occupational	RR = 5.01 (2.38 – 10.57)
Respiratory symptoms	1	Gomzi M. (1995)	Cross – sectional	Environmental	OR = 1.28 (0.51 – 3.206)
Acute Leukemia (Acute Myeloid Leukemia (AML), Acute Promyelocyte Leukemia, Acute Lymphocytic leukemia (ALL), Acute Non-Lymphocytic Leukemia (ANLL))	4	Wong O. (2009)	Case – control	Occupational	OR = 1.64 (1.23 – 2.19) – AML<br><br>OR = 1.41 (0.7 – 2.82) – Acute Promyelocyte Leukemia
		Shi R. (2013)	Case – control	Occupational	OR = 9.5 (1.1 – 79.6) - Childhood
		Wong O. (2010)	Case - control	Occupational	OR = 1.64 (1.23 – 2.19) - AML<br>OR = 1.41 (0.7 – 2.82) – Acute Promyelocyte Leukemia
		Schwartzbaum JA (1991)	Cross – sectional	Environmental	OR = 1.3 (Not Reported) – ALL<br>OR = 0.9 (Not Reported) - ANLL
B-cell neoplasms	1	Wong O. (2010)	Case - control	Occupational	OR = 1.68 (1.25 – 2.27) – all<br><br>OR = 1.62 (0.83 – 3.17) – Precursor B-cell neoplasms<br><br>OR = 1.7 (1.22 – 2.37) – Mature B-cell neoplasms
Chronic Leukemia (Chronic Lymphocytic Leukemia (CLL), Chronic Myeloid Leukemia (CML), Small Lymphocytic Leukemia (SLL))	2	Wong 0 (2010)	Case – control	Occupational	OR = 1.51 (0.63 – 3.64) – CLL/SLL
		Karakosta (2016)	Case – control	Both	OR = 3.83 (1.98 – 7.41) - CML
		Wong 0 (2010)	Case – control	Occupational	OR = 1.53 (1.17 – 2) – NHL<br><br>OR = 0.84 (0.3 – 2.34) – Follicular Lymphoma

Lymphoma (Hodgkin Lymphoma (HL), Non-Hodgkin Lymphoma (NHL))	4				OR = 1.97 (1.27 – 3.06) – Diffuse Large B-cell Lymphoma
		Schwartzbaum JA (1991)	Cross – sectional	Environmental	OR = 1.4 (Not Reported) – HL<br><br>OR = 1.3 (Not Reported) - NHL
		Fluegge (2017)	Ecological study	Not Reported	IRR = 0.98 (0.94 – 1.02) - NHL
		Yiin JH (2016)	Case – control	Occupational	SMR = 0.9 (0.47 – 1.58) - NHL<br><br>SMR = 1.13 (0.14 – 4.09)-HL
Monoclonal gammopathy of undetermined significance (MGUS)	2	Pasqualetti P. (1991)	Case – control	Occupational	OR = 3.13 (1.33 – 7.19)
		Pasqualetti P. (1996)	Case – control	Occupational	OR = 1.57 (1.13 – 2.18)
Multiple myeloma	4	Morris PD (1986)	Case – control	Both	OR = 3 (0.6 - 15.3)
		Cantor KP (1984)	Ecological study	Occupational	OR = 1.7 (1 – 2.9)
		Fluegge (2017)	Ecological study	Not Reported	IRR = 0.99 (0.94 – 1.03)
		Yiin JH (2016)	Exposure Cohort	Occupational	SMR = 1.47 (0.7 – 2.7)
Myelodysplastic syndrome	1	Nisse (2001)	Case – control	Occupational	OR = 2.9 (1.2 – 8)
T/NK- cell neoplasms	1	Wong O. (2010)	Case - control	Occupational	OR =0.74 (0.36 – 1.51)
Leukemia	3	Blair A. (1981)	Ecological study	Occupational	OR = 1.42 (1.06 - 1.92)
		Fluegge (2017)	Ecological study	Not Reported	IRR = 0.95 (0.93 – 0.98)
		Yiin JH (2016)	Exposure Cohort	Occupational	SMR = 1.74 (1.11 – 2.62)
Hematological Malignancies	2	Pasqualetti P. (1991)	Case – control	Occupational	OR = 1.63 (1.09 – 2.43)
		Yiin JH (2016)	Exposure Cohort	Occupational	SMR = 1.34 (0.98 – 1.78)
All Cancer Mortality	3	Bulbuyan (1996)	Exposure Cohort	Occupational	RR = 1.21 (0.81 – 1.81)
		Yiin JH (2016)	Exposure Cohort	Occupational	SMR = 1.16 (1.06 – 1.28)
		Fandrem SI (1993)	Exposure Cohort	Occupational	SMR = 0.95 (0.81 – 1.09)
Brain tumors (glioma, meningioma, neuroblastoma)	3	Musicco M. (1988)	Case – control	Occupational	RR = 1.4 (0.8 – 2.49) - Gliomas
		Efird JT (2003)	Case – control	Occupational	OR = 1.9 (1.3 – 2.8) - childhood
		Schwartzbaum JA (1991)	Cross – sectional	Environmental	OR = 1.1 (Not Reported) - Neuroblastoma
		Yiin JH (2016)	Exposure Cohort	Occupational	SMR = 0.97 (0.44 – 1.83)
		Musicco (1982)	Case – control	Occupational	RR = 12.4, p = 0.011
Tongue cancer	1	Yiin JH (2016)	Exposure Cohort	Occupational	SMR = 0.5 (0.01 – 2.77)
Buccal cavity and pharynx Cancer	1	Yiin JH (2016)	Exposure Cohort	Occupational	SMR = 0.68 (0.25 – 1.48)

Billiary, liver and gall bladder cancer	1	Yiin JH (2016)	Exposure Cohort	Occupational	SMR = 1.16 (0.64 – 1.95)
Urinary tract cancer (Renal, bladder)	3	García-Pérez (2016)	Case – control	Environmental	OR = 3.33 (0.58 – 19.11) - Renal
		Yiin JH (2016)	Exposure Cohort	Occupational	SMR = 1.24 (0.78 – 1.88) – urinary tract<br><br>SMR = 1.28 (0.66 – 2.23) – Renal<br><br>SMR = 1.24 (0.86 – 1.74)- Prostate<br><br>SMR = 1.2(0.57 – 2.2)- Bladder
		Fandrem SI (1993)	Exposure Cohort	Occupational	SMR = 1.18 (0.54 – 2.25)- Renal<br><br>SMR = 0.96 (0.51 – 1.64) Bladder
Breast cancer	1	Yiin JH (2016)	Exposure Cohort	Occupational	SMR = 0.64 (0.13 – 1.86)
Gastric cancer	5	Cocco P. (1994)	Case – control	Occupational	OR = 1.13 (0.94 – 1.36)
		Cocco P. (1999)	Case – control	Occupational	OR = 1 (0.81 – 1.24)
		Bulbuyan (1996)	Exposure Cohort	Occupational	SMD, p=0.08
		Yiin JH (2016)	Exposure Cohort	Occupational	SMR = 0.91 (0.44 – 1.68)
		Fandrem SI (1993)	Exposure Cohort	Occupational	SMR = 0.89 (0.49 – 1.45)
Digestive system and peritoneum cancer	1	Yiin JH (2016)	Exposure Cohort	Occupational	SMR = 0.91 (0.73 – 1.13)
Esophagus cancer	2	Yiin JH (2016)	Exposure Cohort	Occupational	SMR = 0.99 (0.53 – 1.7)
		Fandrem SI (1993)	Exposure Cohort	Occupational	SMR = 0.42 (Not Reported)
Rectum cancer	2	Yiin JH (2016)	Exposure Cohort	Occupational	SMR = 0.32 (0.04 – 1.14)
		Fandrem SI (1993)	Exposure Cohort	Occupational	SMR = 0.88 (0.56 – 1.33)
Intestine cancer (except rectum)	1	Yiin JH (2016)	Exposure Cohort	Occupational	SMR = 0.86 (0.55 – 1.26)
Pancreatic cancer	2	Yiin JH (2016)	Exposure Cohort	Occupational	SMR = 0.98 (0.59 – 1.53)
		Fandrem SI (1993)	Exposure Cohort	Occupational	SMR = 1.23 (0.56 – 2.34)
Colon cancer	1	Fandrem SI (1993)	Exposure Cohort	Occupational	SMR = 0.88 (0.56 – 1.33)
Lung cancer	3	Menvielle G. (2003)	Case – control	Occupational	OR = 1.2 (0.5 – 3.3)
		Bulbuyan (1996)	Exposure Cohort	Occupational	SMD, p=0.98
		Fandrem SI (1993)	Exposure Cohort	Occupational	SMR = 1.09 (0.73 – 1.53)

Germ cell cancer	1	Schmeisser N. (2011)	Case – control	Occupational	OR = 1 (0.4 – 2.4)
Cancer of other parts of buccal cavity	1	Yiin JH (2016)	Exposure Cohort	Occupational	SMR = 0.96 (0.12 – 3.47)
Pharynx cancer	1	Yiin JH (2016)	Exposure Cohort	Occupational	SMR = 0.64 (0.13 – 1.88)
Respiratory system cancer	1	Yiin JH (2016)	Exposure Cohort	Occupational	SMR = 1.3 (1.11 – 1.51)
Larynx cancer	1	Yiin JH (2016)	Exposure Cohort	Occupational	SMR = 1.08 (0.35 – 2.52)
Uveal Melanoma	1	Behrens T. (2011)	Case – control	Occupational	OR = 8.93 (1.73 – 42.13)
Wilm's Tumor	1	Schwartzbaum JA (1991)	Cross – sectional	Environmental	OR = 0.7 (Not Reported)
Osteosarcoma	1	Schwartzbaum JA (1991)	Cross – sectional	Environmental	OR = 2.6 (Not Reported)
Ovarian cancer mortality	1	García-Pérez (2015)	Ecological study	NR	OR = 1.22 (1 – 1.46)
Ewing's sarcoma	2	Holly EA (1991)	Case – control	Occupational	RR = 6.1 (1.7 – 21.9)
		Schwartzbaum JA (1991)	Cross – sectional	Environmental	OR = 1.1 (Not Reported)
Cancer of trachea, bronchus, and lung	1	Yiin JH (2016)	Exposure Cohort	Occupational	SMR = 1.32 (1.13 – 1.53)
Cancer of male genital organs	3	Yiin JH (2016)	Exposure Cohort	Occupational	SMR = 1.34 (0.95 – 1.84)
		Haughey BP (1989)	Case – control	Occupational	OR = 2.27 (1.3 – 5) – Testicular cancer
		Kristensen P. (1996)	Exposure Cohort	Occupational	RR = 1.84 (1.22 – 2.76) – Testicular cancer
Cancer of other male genital organs	1	Yiin JH (2016)	Exposure Cohort	Occupational	SMR = 3.93 (1.07 – 10.1)
Cancer of other and unspecified organs	1	Yiin JH (2016)	Exposure Cohort	Occupational	SMR = 1.21 (0.92 – 1.56)
Skin cancer	2	Yiin JH (2016)	Exposure Cohort	Occupational	SMR = 1.69 (0.9 – 2.89)
		Fandrem SI (1993)	Exposure Cohort	Occupational	SMR = 0.79 (0.25 – 1.83) - Melanoma
Mesothelioma	2	Yiin JH (2016)	Exposure Cohort	Occupational	SMR = 0.9 (0.02 – 5)
		Fandrem SI (1993)	Exposure Cohort	Occupational	SMR Not reported (Cancer of Pleura)
Connective tissue cancer	1	Yiin JH (2016)	Exposure Cohort	Occupational	SMR = 0.96 (0.12 – 3.45)
Creutzfeldt-Jakob disease (CJD)	1	Van Duijn (1998)	Case – control	Both	OR = 0.76 (0.43 – 1.36)
Dehydration	1	Khuder (2007)	Cross – sectional	Environmental	Chi^2, p = 0.009
Diabetes Mellitus	1	Yiin JH (2016)	Exposure Cohort	Occupational	SMR = 1.09 (0.77 – 1.48)
Abdominal bloating	1	Khuder (2007)	Cross – sectional	Environmental	Chi^2, p = 0.02
Alcoholism	1	Yiin JH (2016)	Cohort	Occupational	SMR = 0.65 (0.24 – 1.42)
Jaundice	1	Khuder (2007)	Cross – sectional	Environmental	Chi^2, p = 0.0012
Narcolepsy	1	Ton TGN (2010)	Case – control	Occupational	OR = 1.8 (0.7 – 4.6)
Polydactyly	1	Kristensen P. (1997)	Cohort	Occupational	OR = 1.54 (0.98 – 2.42)

Esopgageal atresia	1	Kristensen P. (1997)	Cohort	Occupational	OR = 1.99 (0.97 – 4.08)
Low birth weight	1	Wang N. (2018)	Case – control	Environmental	OR = 2.51 (1.05 – 5.99)
Syndactyly	1	Kristensen P. (1997)	Cohort	Occupational	OR = 1.85 (1.15 – 2.99)
Rheumatoid Arthritis	1	Parks C. (2016)	Cohort	Occupational	OR = 1.7 (1.1 – 2.7)
Skin conditions (rash / lesions/ ulcer)	3	Gorman Ng M (2013)	Case – control	Occupational	OR = 2.18 (0.98 – 4.85) – skin rash
		Melkonian (2010)	Cohort	Occupational	HR = 1.97 (1.65 – 2.35) – skin lesions
		Khuder (2007)	Cross – sectional	Environmental	Chi^2, p = 0.035 - skin ulcer
Tear secretion	1	Khuder (2007)	Cross – sectional	Environmental	Chi2, p = 0.023
Vascular Dementia	1	Lindsay J. (1997)	Population-based case-control	Occupational	OR = 2,6 (1.3 – 5.23)
Amyotrophic Lateral Sclerosis (ALS)	1	Yu Y. (2014)	Case – control	Both	OR = 2.97 (0.81 – 10.9)
Weekness	1	Khuder (2007)	Cross – sectional	Environmental	Chi^2, p = 0.043
All-cause mortality	1	Yiin JH (2016)	Cohort	Occupational	SMR = 1.07 (1.02 – 1.13)
Aplastic Anemia	1	Issaragrisil (2006)	Case – control	Both	RR = 1.1 (Not Reported)
Weight loss	1	Khuder (2007)	Cross – sectional	Environmental	Chi^2, p = 0.001

* HR = hazard ratio, OR = odds ratio, RR = risk ratio, SMR = standardized mortality ratio.

Supplementary Table S3. Studies associated with exposure to organic fertilizers and health outcomes.

Health outcome	Number of studies for each health outcome	First author (year of publication)	Study type	Type of exposure (occupational / environmental / both)	Outcome metrics
Acute Bronchitis	1	Khuder (2007)	Cross-sectional	Environmental	b = -0.29
Asthma	1	Illi (2012)	Cohort	Environmental	OR = 0.65 (0.47 – 0.9)
Chest congestion	1	Dorn CR (1985)	Cohort	Occupational	OR = 1.98 (Not Reported)
Chronic Bronchitis	1	Valcin M (2007)	Cross-sectional	Occupational	OR = 1.58 (1.18 – 2.12)
Combined lower respiratory symptoms	1	Dorn CR (1985)	Cohort	Occupational	OR = 0.72 (Not Reported)
Combined upper respiratory symptoms	1	Dorn CR (1985)	Cohort	Occupational	OR = 1.21 (Not Reported)
Cough	1	Sinh Dang – Xuan (2017)	Cross – sectional	Both	OR = 1.22 (0.85 – 1.74)
Hay Fever	1	Illi (2012)	Cohort	Environmental	OR = 0.51 (0.33 – 0.8)
Emphysema	1	Khuder (2007)	Cross – sectional	Environmental	Chi^2, p = 0.025
Nasal congestion	1	Dorn CR (1985)	Cohort	Occupational	OR = 2 (Not Reported) – First sludge application<br><br>OR = 12.11 (Not Reported) – Second sludge application
Runny nose	1	Dorn CR (1985)	Cohort	Occupational	OR = 0.9 (Not Reported) – First sludge application<br><br>OR = 0.69 (Not Reported) – Second sludge application
Sore throat	1	Dorn CR (1985)	Cohort	Occupational	OR = 0.83 (Not Reported) – First sludge application<br><br>OR = 1.05 (Not Reported) – Second sludge application
Any of: Fever, Headache, GMAP, Nausea, Diarrhea, Runny nose, Sore throat, Nasal congestion, Hoarseness, Chest congestion, Cough	1	Dorn CR (1985)	Cohort	Occupational	OR = 1.13 (Not Reported)
Aplastic anemia	1	Issaragrisil (2006)	Case – control	Occupational	RR = 2.1 (1 – 4.4)

Atopic dermatitis	1	Illi (2012)	Cohort	Environmental	OR = 0.66 (0.45 – 0.96)
Atopic sensitization	1	Illi (2012)	Cohort	Environmental	OR = 0.85 (0.65 – 1.11)
Skin ulcer	1	Khuder (2007)	Cross – sectional	Environmental	Chi^2, p = 0.035
Rheumatoid Arthritis	1	Parks C. (2016)	Cohort	Occupational	OR = 0.85 (0.48 – 1.5)
Abdominal bloating	1	Khuder (2007)	Cross – sectional	Environmental	Chi^2, p = 0.02
Jaundice	1	Khuder (2007)	Cross – sectional	Environmental	Chi^2, p = 0.012
Combined digestive symptoms	1	Dorn CR (1985)	Cohort	Occupational	OR = 2.69 (Not Reported)
Diarrhea / nausea / vomiting	4	Dorn CR (1985)	Cohort	Occupational	OR = 4.42 (Not Reported) - Diarrhea<br><br>OR = 6.04 (Not Reported) - Nausea
		Pham-Duc (2014)	Nested case - control	Environmental	OR = 1.6 (1 – 2.6)
		Sinh Dang-Xuan (2017)	Cross – sectional	Both	OR = 0.99 (0.35 – 2.82)
		Contreras JD (2020)	Bayesian spatial analysis	Environmental	RR = 0.3 (0.11 – 0.82) (100-fold increase in distance from the canal)
Combined general symptoms	1	Dorn CR (1985)	Cohort	Occupational	OR = 1.55 (Not Reported)
Community-acquired MRSA	1	Casey (2013)	Nested case – control	Both	OR = 1.38 (1.13 – 1.69) – swine manure<br><br>OR = 1.24 (1.01 – 1.52) – Dairy / veal manure
Creutzfeldt-Jakob disease (CJD)	1	Van Duijn (1998)	Case – control	Both	OR = 2.32 (1.38 – 2.91)
Cutaneous leishmaniasis	1	Robles G. (2018)	Case – control	Environmental	OR = 2.312 (0.514 – 10.4012)
Escherichia coli O157:H7 antibodies	1	Belongia (2003)	Cross – sectional	Environmental	OR = 1.9 (1.2 – 3.2)
Fever	2	Dorn CR (1985)	Cohort	Occupational	OR = 0.85 (Not Reported) – First sludge application<br><br>OR = 1.39 (Not Reported) – Second sludge application
		Sinh Dang-Xuan (2017)	Both	Both	OR = 1.62 (0.97 – 2.71)
Generalized muscle aches and pains	1	Dorn CR (1985)	Cohort	Occupational	OR = 0.34 (Not Reported)

Helminth Infection	1	Pham-Duc (2013)	Cross – sectional	Environmental	OR = 1.3 (0.9 – 2)
Hospital acquired MRSA	1	Casey (2013)	Nested case – control	Both	OR = 1.3 (1.05– 1.61) – swine manure<br><br>OR = 0.88 (0.62 – 0.98) – Dairy / veal manure
Household poultry positive for C. jejuni	1	El-Tras (2005)	Cross – sectional	Occupational	OR = 24.5 (5.58 – 102.8)
Malaria and Soil Transmitted Helminthiasis coinfection	1	Getachew (2013)	Cross – sectional	Environmental	OR = 5.34 (1.99 – 14.28)
Q fever seroprevalence against C. burnetii	1	Dal Pozzo (2015)	Cross – sectional	NR	OR = 6.77 (1.8 – 25.45)
Schistosomiasis	1	Carlton (2015)	Cross – sectional	Environmental	OR = 0.68 (0.37 – 1.25)
Skin/ soft tissue infection	1	Casey (2013)	Nested case – control	Both	OR = 1.37 (1.18– 1.6) – swine manure<br><br>OR = 1.01 (0.87 – 1.19) – Dairy / veal manure
Lung cancer	1	Menvielle G. (2003)	Case – control	Occupational	OR = 1.1 (0.2 – 5.6)
Uveal melanoma	1	Behrens T. (2011)	Case – control	Occupational	OR = 8.11 (1.51 – 43.5)
Tear secretion	1	Khuder (2007)	Cross – sectional	Environmental	Chi^2, p = 0.023
Weakness	1	Khuder (2007)	Cross – sectional	Environmental	Chi^2, p = 0.043
Weight loss	1	Khuder (2007)	Cross – sectional	Environmental	Chi^2, p = 0.001
Dehydration	1	Khuder (2007)	Cross-sectional	Environmental	Chi2, p = 0.009
Headache	1	Dorn CR (1985)	Cohort	Occupational	OR = 1.5 (Not Reported) – First sludge application<br><br>OR = 1.37 (Not Reported) – Second sludge application
Hoarseness	1	Dorn CR (1985)	Cohort	Occupational	OR = 0.45 (Not Reported) – First sludge application<br><br>OR = 0.98 (Not Reported) – Second sludge application

* HR = hazard ratio, OR = odds ratio, RR = risk ratio, SMR = standardized mortality ratio.

Supplementary Table S4. PRISMA-P Checklist.

Section and Topic	Item #	Checklist item	Location where item is reported
**TITLE**			
Title	1	Identify the report as a systematic review.	Line 2
**ABSTRACT**			
Abstract	2	See the PRISMA 2020 for Abstracts checklist.	Line 15
**INTRODUCTION**			
Rationale	3	Describe the rationale for the review in the context of existing knowledge.	Line 51
Objectives	4	Provide an explicit statement of the objective(s) or question(s) the review addresses.	Line 72
**METHODS**			
Eligibility criteria	5	Specify the inclusion and exclusion criteria for the review and how studies were grouped for the syntheses.	Line 115
Information sources	6	Specify all databases, registers, websites, organisations, reference lists and other sources searched or consulted to identify studies. Specify the date when each source was last searched or consulted.	Line 102
Search strategy	7	Present the full search strategies for all databases, registers and websites, including any filters and limits used.	Line 103
Selection process	8	Specify the methods used to decide whether a study met the inclusion criteria of the review, including how many reviewers screened each record and each report retrieved, whether they worked independently, and if applicable, details of automation tools used in the process.	Line 110
Data collection process	9	Specify the methods used to collect data from reports, including how many reviewers collected data from each report, whether they worked independently, any processes for obtaining or confirming data from study investigators, and if applicable, details of automation tools used in the process.	Line 143
Data items	10a	List and define all outcomes for which data were sought. Specify whether all results that were compatible with each outcome domain in each study were sought (e.g. for all measures, time points, analyses), and if not, the methods used to decide which results to collect.	Line 139
	10b	List and define all other variables for which data were sought (e.g. participant and intervention characteristics, funding sources). Describe any assumptions made about any missing or unclear information.	Line 151
Study risk of bias assessment	11	Specify the methods used to assess risk of bias in the included studies, including details of the tool(s) used, how many reviewers assessed each study and whether they worked independently, and if applicable, details of automation tools used in the process.	Line 170
Effect measures	12	Specify for each outcome the effect measure(s) (e.g. risk ratio, mean difference) used in the synthesis or presentation of results.	Line 161
Synthesis methods	13a	Describe the processes used to decide which studies were eligible for each synthesis (e.g. tabulating the study intervention characteristics and comparing against the planned groups for each synthesis (item #5)).	Line 177
	13b	Describe any methods required to prepare the data for presentation or synthesis, such as handling of missing summary statistics, or data conversions.	-
	13c	Describe any methods used to tabulate or visually display results of individual studies and syntheses.	-
	13d	Describe any methods used to synthesize results and provide a rationale for the choice(s). If meta-analysis was performed, describe the model(s), method(s) to identify the presence and extent of statistical heterogeneity, and software package(s) used.	178
	13e	Describe any methods used to explore possible causes of heterogeneity among study results (e.g. subgroup analysis, meta-regression).	-
	13f	Describe any sensitivity analyses conducted to assess robustness of the synthesized results.	-
Reporting bias assessment	14	Describe any methods used to assess risk of bias due to missing results in a synthesis (arising from reporting biases).	-
Certainty assessment	15	Describe any methods used to assess certainty (or confidence) in the body of evidence for an outcome.	-
**RESULTS**			
Study selection	16a	Describe the results of the search and selection process, from the number of records identified in the search to the number of studies included in the review, ideally using a flow diagram.	Line 189

Supplementary Table S4. PRISMA-P Checklist.

Section and Topic	Item #	Checklist item	Location where item is reported
	16b	Cite studies that might appear to meet the inclusion criteria, but which were excluded, and explain why they were excluded.	Line 83
Study characteristics	17	Cite each included study and present its characteristics.	Line 190
Risk of bias in studies	18	Present assessments of risk of bias for each included study.	Line 249 & 384
Results of individual studies	19	For all outcomes, present, for each study: (a) summary statistics for each group (where appropriate) and (b) an effect estimate and its precision (e.g. confidence/credible interval), ideally using structured tables or plots.	Line 249 & 384
Results of syntheses	20a	For each synthesis, briefly summarise the characteristics and risk of bias among contributing studies.	-
	20b	Present results of all statistical syntheses conducted. If meta-analysis was done, present for each the summary estimate and its precision (e.g. confidence/credible interval) and measures of statistical heterogeneity. If comparing groups, describe the direction of the effect.	-
	20c	Present results of all investigations of possible causes of heterogeneity among study results.	-
	20d	Present results of all sensitivity analyses conducted to assess the robustness of the synthesized results.	-
Reporting biases	21	Present assessments of risk of bias due to missing results (arising from reporting biases) for each synthesis assessed.	-
Certainty of evidence	22	Present assessments of certainty (or confidence) in the body of evidence for each outcome assessed.	-
**DISCUSSION**			
Discussion	23a	Provide a general interpretation of the results in the context of other evidence.	Line 473
	23b	Discuss any limitations of the evidence included in the review.	Line 607
	23c	Discuss any limitations of the review processes used.	Line 607
	23d	Discuss implications of the results for practice, policy, and future research.	Line 644
**OTHER INFORMATION**			
Registration and protocol	24a	Provide registration information for the review, including register name and registration number, or state that the review was not registered.	-
	24b	Indicate where the review protocol can be accessed, or state that a protocol was not prepared.	-
	24c	Describe and explain any amendments to information provided at registration or in the protocol.	-
Support	25	Describe sources of financial or non-financial support for the review, and the role of the funders or sponsors in the review.	-
Competing interests	26	Declare any competing interests of review authors.	-
Availability of data, code and other materials	27	Report which of the following are publicly available and where they can be found: template data collection forms; data extracted from included studies; data used for all analyses; analytic code; any other materials used in the review.	-

From: Page MJ, McKenzie JE, Bossuyt PM, Boutron I, Hoffmann TC, Mulrow CD, et al. The PRISMA 2020 statement: an updated guideline for reporting systematic reviews. BMJ 2021;372:n71. doi: 10.1136/bmj.n71
